# Iron, Hepcidin and Inflammatory Status of Young Healthy Overweight and Obese Women in Australia

**DOI:** 10.1371/journal.pone.0068675

**Published:** 2013-07-04

**Authors:** Hoi Lun Cheng, Christian E. Bryant, Kieron B. Rooney, Katharine S. Steinbeck, Hayley J. Griffin, Peter Petocz, Helen T. O’Connor

**Affiliations:** 1 Discipline of Exercise and Sport Science, Faculty of Health Sciences, The University of Sydney, Sydney, New South Wales, Australia; 2 Anzac Research Institute, Concord Campus, The University of Sydney, Sydney, New South Wales, Australia; 3 Academic Department of Adolescent Medicine, The University of Sydney, Sydney, New South Wales, Australia; 4 Department of Statistics, Macquarie University, Sydney, New South Wales, Australia; Penn State Hershey Medical Center, United States of America

## Abstract

**Background and Aims:**

Evidence suggests obesity-related inflammation alters iron metabolism potentially increasing the risk of iron deficiency. This cross-sectional study aimed to investigate iron, hepcidin and inflammatory status in young, healthy overweight and obese women.

**Methods:**

114 young (18–25 years), healthy comorbidity-free women with a body mass index (BMI) ≥27.5 kg/m^2^ were recruited. Biochemical data were analysed using mean ± standard deviation or median (interquartile range) and multivariate modelling. Biochemical markers were also stratified according to varying degrees of overweight and obesity.

**Results:**

Anaemia (haemoglobin <120 g/l) and iron deficiency (serum ferritin <15.0 µg/l) were prevalent in 10% and 17% of participants respectively. Mean/median soluble transferrin receptor was 1.61±0.44 mg/l; hepcidin 6.40 (7.85) ng/ml and C-reactive protein (CRP) 3.58 (5.81) mg/l. Multivariate modelling showed that BMI was a significant predictor of serum iron (coefficient = -0.379; standard error = 0.139; *p* = 0.008), transferrin saturation (coefficient = -0.588; standard error = 0.222; *p* = 0.009) and CRP (coefficient = 0.127; standard error = 0.024; *p*<0.001). Stratification of participants according to BMI showed those with ≥35.0 kg/m^2^ had significantly higher CRP (*p*<0.001) than those in lower BMI categories.

**Conclusions:**

Increasing obesity was associated with minor disturbances in iron metabolism. However, overall outcomes indicated simple iron deficiency (hypoferritinaemia) was the primary iron-related abnormality with no apparent contribution of inflammation or hepcidin, even in those with BMI >35.0 kg/m^2^. This indicates that obesity alone may not be sufficient to induce clinically significant disturbances to iron metabolism as previously described. This may be attributed to the lack of comorbidity in this cohort.

## Introduction

Obesity is regarded as a pro-inflammatory condition characterised by the presence of chronic, low grade systemic inflammation. [1], [2] In the past decade, a growing number of studies have suggested that obesity-related inflammation can lead to an iron handling defect similar to anaemia of inflammation (AI), [1] with hepcidin proposed as a key mediator.[2]–[4] Our recent systematic review investigating the impact of obesity on iron status also reported elevated BMI to be associated with increased serum ferritin and reduced transferrin saturation. [5] However the question of whether these alterations are due to inflammation-mediated functional deficiency or true iron deficiency remains unclear due to lack of data for soluble transferrin receptor (sTfR) and hepcidin. [5].

At a population level, premenopausal women are at higher risk of iron deficiency. In Australia, it has been estimated that over 20% of women between 25–50 years are either iron deficient or have marginally low iron status. [6] In addition to regular menstrual iron loss, [7] low iron intake (which is often reported in this group) and restrictive dietary practices for weight loss in overweight women can also increase the risk of iron deficiency. [8], [9] Overweight and obese young women may therefore have a combination of age, dietary and inflammatory-associated factors perturbing iron status.

Currently, only a handful of dedicated studies on obesity-related iron deficiency have focused on adolescent girls or young women. [3], [10], [11] One US study analysed serum hepcidin and reported it to be significantly higher in bariatric surgery candidates compared to non-obese women. [3] Severe obesity however, is frequently associated with comorbidities (e.g. obstructive sleep apnoea, gastro-oesophageal reflux and fatty liver disease) that influence haematological markers or increase blood loss, [5], [12], [13] and findings from this study may not be applicable to the wider population of less severely obese young women who are not burdened with comorbid conditions. Furthermore, other studies performed in more representative populations have been limited by the absence of hepcidin measurement, with mixed outcomes further complicating interpretation.

This study aimed to clarify the relationship between obesity, inflammation and iron status and identify the prevalence and nature of iron deficiency in a cohort of young healthy overweight and obese women by using a broad range of iron markers including sTfR and hepcidin.

## Materials and Methods

### Ethics Statement

This study was registered with the Australian New Zealand Clinical Trials Registry (ID: ACTRN12613000072718) and approved by the Ethics Review Committee of the Sydney South West Area Health Service and the University of Sydney Ethics Committee. Signed, written informed consent was obtained from all participants. Ethics approval was obtained for the referral of participants to their family physician upon detection of abnormal pathology results and review by the study medical officer (KSS).

### Study Design

This cross-sectional study reports anthropometric and biochemical characteristics of a community sample of young overweight and obese women. Participants included in this study responded to one of two advertisements: one for recruitment into this cross-sectional study and the other for recruitment into a related 12 month clinical weight loss trial (where a similar but more stringent eligibility criteria was enforced). [14] This cross-sectional study reports the baseline characteristics of volunteers who responded to either advertisement and met the eligibility criteria outlined in this manuscript. A subset of these participants also met the recruitment criteria for entry into the weight loss trial and results from this trial have been published elsewhere. [14].

### Participants

Young women aged between 18 to 25 years with a measured BMI ≥27.5 kg/m^2^ were recruited. Exclusion criteria included: self-report of significant medical conditions [e.g. diabetes mellitus, disorders of the liver (including hemochromatosis) and kidney, autoimmune or metabolic diseases and malignancy] or use of medications that may influence weight, iron or inflammatory status; pregnancy or lactation; vegetarianism; zinc supplementation; smoking; and previous bariatric surgery.

Volunteers reporting iron supplementation or blood donation within three months prior to recruitment were asked to participate only following a three month washout period. A two week washout period was required prior to recruitment for self-reported vitamin, fish oil or mineral (other than iron or zinc) supplementation.

### Data Collection

Participants attended two appointments at the obesity clinic of a university teaching hospital in Sydney, Australia. The first visit involved anthropometric assessment and collection of medical and socio-demographic history including self-reported use of contraceptive medication and history of polycystic ovary syndrome (PCOS). Participants reported to the second visit following a 12 hour overnight fast for collection of a morning venous blood sample. Those reporting current or recent (week prior) acute infection had blood collection rescheduled to avoid infection related inflammation.

Height (to the nearest 0.1 cm) and weight (to the nearest 0.1 kg) were measured without shoes using a wall-mounted stadiometer (Hyssna Limfog AB, Hyssna, Sweden) and an electronic digital platform scale (Teraoka DS-260; Teraoka Seiko, Tokyo, Japan) respectively. Waist circumference was measured using a metal retractable tape (Lufkin W606PM; Cooper Industries, Sparks, MD) to the nearest 0.1 cm and according to international guidelines. [15] In participants who were subsequently recruited to the weight loss trial, baseline body composition was also measured by dual-energy x-ray absorptiometry (DXA) (GE Lunar Prodigy; GE Healthcare, Chalfont St Giles, UK). Abdominal fat was calculated by taking the lower boundary at the pelvis cut; upper boundary above the pelvis cut by 20% of the distance between the pelvis and neck cuts; and lateral boundaries at the arm cuts.

### Biochemical Analysis

Haemoglobin, serum iron, transferrin saturation and serum ferritin were analysed at a nationally accredited commercial diagnostic pathology laboratory. Stored frozen plasma was used to analyse sTfR, hepcidin and C-reactive protein (CRP). Haemoglobin was measured using the Sysmex instrument (Roche Diagnostics Australia, Sydney, Australia). Serum iron, transferrin saturation and serum ferritin were measured using the Roche Modular E170 immunoassay analyser (Roche Diagnostics Australia, Sydney, Australia). The reference ranges used were 120–165 g/l for haemoglobin; 10.0–30.0 µmol/l for serum iron; 12.0–45.0% for transferrin saturation; and 15.0–165.0 µg/l for serum ferritin. Plasma sTfR and CRP were analysed using commercial ELISA kits (R&D Systems, Minneapolis, MN). Reference ranges provided were 0.74–2.39 mg/l for sTfR; and 0.11–4.52 mg/l for CRP. A conversion factor was applied to transform sTfR values from nmol/l to mg/l [16]. The sTfR-ferritin index (sTfR-F) was used to identify participants with iron depletion (sTfR-F 1.80–2.20), iron deficient erythropoiesis (sTfR-F >2.20), and AI (sTfR-F <1.00 with concurrent haemoglobin <120 g/l). [17], [18] Clinically elevated CRP was defined as >10.00 mg/l. [19] Plasma hepcidin was measured by an on-line extraction coupled to liquid chromatography-tandem mass spectrometry method using the Xevo TQ MS (Waters Corporation, Milford, MA). [20] Inter-assay accuracy was 95% with a coefficient of variation of 8.2%. Assay sensitivity was 2.00 ng/ml, and values below the detectable range were defined as 1.00 ng/ml. A reference range of 1.92–32.40 ng/ml was used. [21], [22].

### Statistical Analysis

Statistical analyses were performed using IBM SPSS Statistics 19 for Windows (IBM Corporation, Armonk, NY, USA). Data are presented as percentages, mean ± standard deviation (SD) or median (interquartile range).

The relationship between obesity, iron and inflammatory status was assessed using two multivariate multiple regression models via MANOVA. Pearson’s correlations between BMI and waist circumference as well as between BMI and the DXA variables (total body fat, absolute fat mass and abdominal fat percentage) were assessed prior to running the models. The first regression model included BMI (as a representative anthropometric variable) and potential confounders [use of contraceptive medication, ethnicity (coded dichotomously as European or non-European) and PCOS] as predictors for the response variables: haemoglobin, serum iron, transferrin saturation, serum ferritin, sTfR, sTfR-F, hepcidin and CRP. As DXA body composition measurement was only conducted in a subset of participants, total body fat was excluded from initial multivariate analysis to maximise the number of participants included in the first regression model. A second multivariate model including BMI, total body fat percentage and the aforementioned confounder variables as predictors was subsequently produced to evaluate the impact of body composition on the biochemical markers. Significance was set at 0.01 for the MANOVA models to protect against type 1 error with regression coefficients and standard errors (SE) also reported.

Secondary analyses were carried out to investigate biochemical differences between varying degrees of overweight and obesity by stratifying BMI into three categories and comparing biochemical markers using one way ANOVA and Bonferroni-adjusted post hoc tests. For the regression models and secondary analyses, variables were assessed for normality with natural log transformations performed on the serum ferritin, sTfR-F, hepcidin and CRP variables. Significance for the post hoc tests was set at 0.05.

## Results

A total of 114 overweight and obese women were recruited to this cross-sectional study. A summary of participant characteristics is presented in Table 1. Mean age and BMI were 22.3±2.3 years and 33.7±4.4 kg/m^2^ respectively. Overall use of hormonal contraceptive medication was 32% (*n* = 36) [oral contraceptive pill (*n* = 30), contraceptive implant (*n* = 4), depot injection (*n* = 1) or patch (*n* = 1)]. PCOS was self-reported in 12% (*n* = 15) of participants. DXA body composition information was obtained from a subset (*n* = 69) of participants (Table 1).

**Table 1:** Summary of participant characteristics.

Participant characteristic (*n* = 114)	Mean ± SD or percentage
Age (years)	22.3±2.3
Ethnicity (%)	
European	65.8%
Asian	12.3%
African	3.5%
South American	4.4%
Other	14.0%
Contraceptive medication (%)	31.6%
PCOS (%)	12.4%
Weight (kg)	94.6±13.3
BMI (kg/m^2^)	33.7±4.4
Waist circumference (cm)	93.8±10.0
Total body fat (%)^a^	49.8±4.5
Absolute fat mass (kg)^a^	44.5±7.6
Abdominal fat (%)^a^	55.7±4.3

SD, standard deviation; PCOS, polycystic ovary syndrome.

a Body composition data obtained from 69 participants. Two participants were precluded from body composition analysis as the measurement equipment did not accommodate body weights of above 130 kg.

A summary of participant iron, hepcidin and inflammatory status is presented in Table 2. Anaemia (haemoglobin <120 g/l) was detected in 10%; (*n* = 11) of participants with none having AI (sTfR-F <1.00). Iron deficiency anaemia was identified in seven of the anaemic participants who simultaneously had serum ferritin and/or transferrin saturation below normal range. The degree of anaemia was mild (107–114 g/l) in the remaining four participants, three of which had borderline to low mean corpuscular volume. Microcytosis with normal iron parameters raises the possibility of thalassemia trait. Pathology results outside of the reference range and which were deemed of potential clinical significance by the study medical officer (KSS) were forwarded to the participant's family physician with their consent.

**Table 2:** Summary of participant biochemistry.

Biochemical marker	Mean ± SD or Median (IQR)	Prevalence of abnormality
Hb (g/l)	131±8	
Hb <120 g/l		9.6%
Serum iron (µmol/l)	15.3±6.8	
Serum iron <10.0 µmol/l		21.9%
Tsat (%)	22.5±10.6	
Tsat <12.0%		14.0%
Serum ferritin (µg/l)	34.0 (37.0)	
Serum ferritin <15.0 µg/l		16.7%
sTfR (mg/l)	1.61±0.44	
sTfR >2.39 mg/l		6.1%
sTfR-F	1.02 (0.65)	
sTfR-F 1.80–2.20		6.1%
sTfR-F >2.20		7.0%
sTfR-F <1.00+ Hb <115 g/l		0.0%
Hepcidin (ng/ml)	6.40 (7.85)	
Hepcidin >32.40 ng/ml		0.9%
CRP (mg/l)	3.58 (5.81)	
CRP>10.00 mg/l		14.0%

SD, standard deviation; IQR, interquartile range; Hb, haemoglobin; Tsat, transferrin saturation; sTfR, soluble transferrin receptor; sTfR-F, soluble transferrin receptor-ferritin index; CRP, C-reactive protein.

Iron deficiency without anaemia (serum ferritin <15.0 µg/l) was present in just under 17% (*n* = 19) of participants. As serum ferritin has been reported to be elevated in obesity, [5] sTfR and sTfR-F were also used to assess iron status. By using elevated sTfR (>2.39 mg/l), 6% of participants were identified as iron deficient. Alternatively, when sTfR-F >1.80 was used, iron deficiency was present in 13% of the cohort. Individuals identified as iron deficient by these alternative methods were also identified by hypoferritinaemia alone.

Median hepcidin was 6.40 ng/ml with only one participant presenting with hepcidin (37.40 ng/ml) above the reference range (in this participant, all other biochemical measures of iron and inflammatory status were normal). Hepcidin concentration was below 5.00 ng/ml in 80% of iron deficient participants. Median CRP (3.58 mg/l) was within the assay specified normal range. Clinically elevated CRP (>10.00 mg/l) was observed in 14% of participants.

Multivariate multiple linear regression models were employed to assess the relationship between obesity, iron and inflammatory status. BMI and total body fat percentage were selected as representative predictors for the anthropometric and body composition measures respectively due to strong correlations between BMI and waist circumference (Pearson’s r = 0.839) and between BMI and each DXA variable (all Pearson’s r>0.737). In the initial regression model (Table 3), BMI was a significant predictor of serum iron (coefficient = -0.379; SE = 0.139; *p* = 0.008), transferrin saturation (coefficient = -0.588; SE = 0.222; *p* = 0.009) and CRP (coefficient = 0.127; SE = 0.024; *p*<0.001). Following the inclusion of total body fat percentage as an additional predictor, the association between BMI and CRP remained (coefficient = 0.149; SE = 0.042; *p* = 0.001), with no significant relationship observed between the DXA and iron variables. Use of contraceptive medication was a significant confounder of iron but not hepcidin status in both regression models and its confounding effect on the first model is shown in Table 3.

**Table 3:** Results from the first multivariate model used to assess the associations between BMI, inflammation, iron and hepcidin status.

Predictor variable	Wilks’ lambda *p* value	Response variable *p* value							
		Hb	Serum iron	Tsat	Serum ferritin^a^	sTfR	sTfR-F^a^	Hepcidin^a^	CRP^a^
BMI	<0.001	0.907	0.008	0.009	0.039	0.833	0.264	0.318	<0.001
Contraception	<0.001	0.600	<0.001	0.006	0.134	0.002	0.007	0.147	<0.001
Ethnicity	0.026	0.124	0.417	0.161	0.553	0.011	0.196	0.723	0.914
PCOS	0.682	0.361	0.957	0.759	0.335	0.602	0.361	0.412	0.288
*R^2^*		0.032	0.181	0.140	0.083	0.148	0.108	0.043	0.347

BMI, body mass index; PCOS, polycystic ovary syndrome; Hb, haemoglobin; Tsat, transferrin saturation; sTfR, soluble transferrin receptor; sTfR-F, soluble transferrin receptor-ferritin index; CRP, C-reactive protein.

a Natural log transformation performed on the serum ferritin, sTfR-F, hepcidin and CRP variables.

Secondary analyses of biochemical data stratified according to World Health Organization BMI classifications were also performed to examine iron, hepcidin and inflammatory status with increasing degrees of overweight and obesity (Table 4). [23] Classes II (35.0–39.9 kg/m^2^) and III (≥40.0 kg/m^2^) obesity were combined into a single category (defined as ≥35.0 kg/m^2^) as the number of participants with higher levels of obesity were small, and this BMI threshold was reported to be associated with substantial iron disturbances in our recent systematic review. [5] Serum iron (*p* = 0.029), transferrin saturation (*p* = 0.042), ferritin (*p* = 0.021) and CRP (*p*<0.001) were significantly different between BMI categories, with CRP showing a significant increment between categories two and three (*p*<0.001) in the post hoc analysis (Table 4).

**Table 4:** Biochemical differences between varying categories of overweight and obesity.

BMICategory	BMI(kg/m^2^)	*n*	Age(years)	Biochemical marker							
				Hb	Serum iron	Tsat	Serum ferritin^a^	sTfR	sTfR-F^a^	Hepcidin^a^	CRP^a^
				(g/l)	(µmol/l)	(%)	(µg/l)	(mg/l)		(ng/ml)	(mg/l)
1	27.5–29.9	28	22.0±2.1	132±8	17.2±6.3	25.9±11.3	31.0 (34.0)	1.64±0.47	1.03 (0.68)	5.25 (8.28)	1.62 (4.15)
2	30.0–34.9	48	21.7±2.3	130±10	16.0±7.4	22.9±10.9	30.5 (31.0)	1.61±0.49	1.02 (0.80)	6.30 (7.70)	1.62 (4.15)
3	≥35.0	38	23.1±2.3	130±8	13.0±6.0^b^	19.3±9.1^b^	46.0 (49.0)^b^	1.58±0.35	0.99 (0.43)	9.20 (9.78)	6.24 (8.17)b,c

Mean ± SD or median (interquartile range).

BMI, body mass index; Hb, haemoglobin; Tsat, transferrin saturation; sTfR, soluble transferrin receptor; sTfR-F, soluble transferrin receptor-ferritin index; CRP, C-reactive protein.

a Natural log transformation performed on the serum ferritin, sTfR-F, hepcidin and CRP variables.

b Biochemical concentration significantly different from BMI category 1 in Bonferroni-adjusted post hoc analysis (*p*<0.05).

c Biochemical concentration significantly different from BMI category 2 in Bonferroni-adjusted post hoc analysis (*p*<0.05).

## Discussion

This study describes iron status in a cohort of healthy overweight and obese young women by using a broad range of iron biomarkers. Unlike studies in older or more severely obese populations, selection of this cohort allowed for examination of obesity, inflammation and iron handling associations independent of potential confounding from significant comorbidity. Young overweight women are of specific interest as they are at risk of iron deficiency. [9] As suboptimal iron status even in the absence of anaemia is reported to adversely affect physical performance, mental health and cognitive function,[24]–[26] success with dietary and behavioural weight management programmes may be reduced secondary to the effects of iron deficiency. [5] Adverse effects from maternal iron deficiency anaemia on birth outcomes, [24] offspring cognitive development, [24] and increase risk for post-partum depression also highlight the importance of iron adequacy in this population. [27].

Results from this study indicate the presence of inflammation and mild iron disruptions such as reduced serum iron and transferrin saturation with increasing BMI. No cases of AI were identified in our participants which is in line with data from the US National Health and Nutrition Examination Survey III. [1] Contrary to our expectations however, simple iron deficiency (hypoferritinaemia) was the most common iron-related abnormality (17%).

Median hepcidin in this cohort was well below the concentration reported in morbidly obese bariatric surgery candidates in the US (88.02 ng/ml). [3] Our hepcidin data was in fact, consistent with the non-obese (BMI <27.0 kg/m^2^) control group of this US study as well as levels reported in healthy, non-anaemic premenopausal female blood donors. [3], [28] The lowest hepcidin concentrations were observed in those with the lowest iron stores. This is consistent with normal suppression of hepatic hepcidin secretion in the iron depleted state to allow for greater iron absorption and mobilisation. [29] It is likely that low iron intake was a major contributing factor to the high prevalence of simple iron deficiency observed, [9] although this cannot be confirmed as dietary iron intake was not assessed. Nevertheless, there was no apparent contribution of obesity-related inflammation to the prevalence of iron deficiency observed as evidenced by the modest rate (14%) of clinically elevated CRP, low median hepcidin and failure of sTfR and sTfR-F to detect a greater percentage of participants with iron deficiency.

Iron but not hepcidin status of young female military recruits has been reported previously. [10] This study concluded that a critical level of adiposity may be required for significant inflammation, hepcidin elevation and iron disturbances to occur which was partially supported by this study. [10] In our subset of participants with a higher degree of obesity (BMI >35.0 kg/m^2^), mean hepcidin concentration remained in the lower normal range despite significantly greater CRP compared to those with lower BMI. Given the healthy state of our cohort, this indicates that a greater level of comorbidity (which is more often seen in older and severely obese individuals) may also be required to elicit significant hepcidin elevation.

This study is primarily limited by the absence of dietary iron intake assessment. As baseline data collection procedures were primarily intended for screening and recruitment into the weight loss trial, assessment of dietary intake was only conducted upon commencement of the weight loss intervention. Iron intake variation however, is not likely to have affected the main finding of this study which was that obesity-related inflammation in the absence of comorbidity did not elicit significant hepcidin-mediated iron disruptions. Furthermore, body composition was only measured in a subset of participants and this may have compromised statistical significance of regression outcomes between the DXA and iron variables.

This study adds to the body of literature on excess weight, iron and inflammatory status in young women inclusive of the markers sTfR, sTfR-F and hepcidin. Results support a positive relationship between obesity and inflammation, with mild disruptions to some iron markers such as serum iron and transferrin saturation. However, simple iron deficiency as reflected by low ferritin remains the major iron-related abnormality in this cohort. There was no apparent contribution of inflammation and hepcidin to iron deficiency prevalence, which may be attributed to the absence of comorbidity in this sample. As true iron deficiency has been shown to compromise reproductive and mental health, cognitive function and general wellbeing, this study highlights the importance of addressing simple iron deficiency in healthy overweight and obese young women.
